# Effects of a community scorecard on improving the local health system in Eastern Democratic Republic of Congo: qualitative evidence using the most significant change technique

**DOI:** 10.1186/s13031-015-0055-4

**Published:** 2015-09-03

**Authors:** Lara S. Ho, Guillaume Labrecque, Isatou Batonon, Viviana Salsi, Ruwan Ratnayake

**Affiliations:** International Rescue Committee, 1730 M Street, Suite 505, Washington, DC 20036 USA; International Rescue Committee Nairobi, P.O. Box 62727-00200, Galana Plaza 4th Floor, Galana Road, Nairobi, Kenya; International Rescue Committee Islamabad, House 11 Street 4 F6/3, Islamabad, Pakistan; International Rescue Committee New York, 122 E 42nd Street, New York, NY 10168 USA

**Keywords:** Community scorecard, Democratic Republic of Congo, Social accountability, Community participation, Fragile and conflict-affected health systems, Most significant change, Health committee

## Abstract

**Background:**

More than a decade of conflict has weakened the health system in the Democratic Republic of Congo and decreased its ability to respond to the needs of the population. Community scorecards have been conceived as a way to increase accountability and responsiveness of service providers, but there is limited evidence of their effects, particularly in fragile and conflict-affected contexts. This paper describes the implementation of community scorecards within a community-driven reconstruction project in two provinces of eastern Democratic Republic of Congo.

**Methods:**

Between June 2012 and November 2013, 45 stories of change in the health system were collected from village development committee, health committee, community members (20 men and 18 women) and healthcare providers (*n* = 7) in 25 sites using the Most Significant Change technique. Stories were analyzed qualitatively for content related to the types and mechanisms of change observed.

**Results:**

The most salient changes were related to increased transparency and community participation in health facility management, and improved quality of care. Quality of care included increased access to services, improved patient-provider relationships, improved performance of service providers, and improved maintenance of physical infrastructure. Changes occurred through many different mechanisms including provider actions in response to information, pressure from community representatives, or supervisors; and joint action and improved collaboration by health facility committees and providers.

**Conclusions:**

Although it is often assumed that confrontation is a primary mechanism for citizens to change state-provided services, this study demonstrates that healthcare providers may also be motivated to change through other means. Positive experiences of community scorecards can provide a structured space for interface between community members and the health system, allowing users to voice their opinions and preferences and bridge information gaps for both users and frontline healthcare providers. When solutions to problems identified through the scorecard are locally accessible, users and healthcare providers are able to work together to implement mutually acceptable solutions that improve quality of health services, and make them more responsive to users’ needs.

## Background

More than a decade of conflict has weakened the already fragile health system in the Democratic Republic of Congo (DRC) and decreased its ability to respond to the needs of the population. In 2013, the DRC ranked last out of 168 countries on the Human Development Index [1]. With 74 % of the population living more than five kilometers from a health center [2], health services are often inaccessible and essential inputs such as drugs and personnel are often unavailable. Lack of funds, along with poor financial management and corruption, have led to a reliance on high user fees and unofficial payments by users to help cover staff salaries, operational costs, and health zone management. The majority of households have difficulty paying for healthcare [3]. This translates into low utilization rates [3].

As in many fragile or conflict-affected states, there is a weak culture of accountability in DRC, characterized by the absence of a social contract between citizens and the state [4, 5]. In the health system, there are few mechanisms through which healthcare providers and the Ministry of Public Health (MoPH) can be held accountable, answer questions from users, or be sanctioned. Weak management and absent accountability relationships lead to corruption, lack of motivation, absenteeism, poor planning, and inadequate implementation of health services and policies [6]. There is limited budget transparency, making it difficult for citizens to hold the government accountable for spending [7]. Communities, and at times service providers themselves, lack information about national health standards, entitlements, and performance, which limits their capacity to monitor service delivery and healthcare providers’ performance. There are few structured and non-partisan spaces for users to dialogue with healthcare providers; in addition, mechanisms to address grievances are rare and often seen as ineffective. Health facility committees can serve as a vehicle for user feedback and demands for service improvements [8], but in the DRC these structures are rarely functional or have often been co-opted by service providers. In this context, efforts to strengthen accountability of healthcare providers to users are seen as important given their potential to increase access and improve service quality [9].

Since 2007 the International Rescue Committee (IRC) and its partner CARE International, have implemented a large-scale community-driven reconstruction project in eastern DRC, named *Tuungane* (“Let’s Unite” in Kiswahili). The aim of the project is to ensure that community priorities and well-being are sustainably supported by a capable and accountable local governance system. The theory of change postulates that people’s needs are best met when public authorities are capable of providing basic services, when they are responsive to citizens’ needs and priorities, and when the general public can engage in decision making and hold them to account. *Tuungane* is currently implemented in 1,025 communities across four provinces (Katanga, Maniema, North Kivu, and South Kivu) and had reached 2.6 million people by December 2014.

In 2011, *Tuungane* revised its implementation strategy to ensure greater sustainability through a focus on service delivery and engagement with existing user committees as well as local authorities. As part of this effort, *Tuungane* introduced the Community Scorecard (CSC) for communities that chose to invest in the education or health sector. Community scorecards were developed to increase accountability and responsiveness to users [10] by providing a space for dialogue between users and service providers, with the goal of improving service delivery. In fragile and conflict-affected contexts where existing levels of trust and accountability may be low, CSC may have the potential to make even greater gains, or alternately may be limited by a lack of confidence and willingness of communities to engage with state institutions [11]. However, as with many social accountability interventions, there is limited evidence of the impact of CSC on the quality and accessibility of services, and on which factors contribute to their success [12]. Even less is known about the effects of social accountability tools in fragile and conflict-affected contexts. Gaventa and McGee (2013) note that “[a] number of good, specific studies exist, using a range of methods, but there are [currently] not enough of these, across enough settings and methods, to begin to point unequivocally to overall patterns or to draw higher-order conclusions” [13].

This paper describes the implementation of a community scorecard approach for the health sector in Katanga and South Kivu provinces in eastern DRC and participants’ perspectives on how it affected service delivery within the local health system as documented by stories collected using the Most Significant Change (MSC) technique, a form of participatory monitoring and evaluation.

## Methods

### Setting

Communities included in *Tuungane* were rural, with populations ranging from 350 to 8,750. Most inhabitants who are not service providers (i.e. teachers or healthcare workers) depend on agriculture for their livelihoods. The majority of health facilities in target communities were health posts, under the supervision of a health center staffed by at least one skilled nurse, as well as auxiliary staff. The remaining structures were health centers staffed which should be staff by multiple skilled staff. Skilled staff in the targeted health structures are likely to come from other parts of the province, but auxiliary staff are most likely to be local staff from the community. Each community involved was made up of one to three villages participating in *Tuungane*.

A “CSC site” is a health facility and the community engaged in the CSC process targeting that facility (1–3 villages). The sampled CSC sites were located in South Kivu province (Minova and Kalehe health zones) and Katanga province (Kambove, Kapalowe, and Kilela Balanda health zones). These areas were targeted because they included both conflict-affected and more stable areas; were accessible to project staff and had completed at least the baseline scorecard in the CSC process at the time of the launch of the MSC exercise.

### The Tuungane process

Community members in each of the 1,025 communities were informed that they would receive a 24,000 USD grant for investment in one of five sectors of their choosing: health, education, roads, markets, and water & sanitation. These sectors were pre-selected because of the technical capacities of the implementing organizations to ensure effective support to community project implementation. *Tuungane* staff facilitated a general assembly to identify a priority sector for investment. At least 20 % of the community attended, and staff ensured women, men, elders, girls, boys, and vulnerable populations were represented. One hundred and fifty-one (15 % or 151/1,025) communities chose to invest their funds in the health sector.

Once the priority sector had been identified, each community elected a Village Development Committee (VDC) to manage implementation of the infrastructure and service improvement project. VDCs are composed of a president, vice-president, secretary, treasurer, and community mobilizer. Women must hold two or three of these five positions to ensure a gender balance. There were five criteria to run for elections: not being a civil servant or a village chief, not being a member of the health facility committee (HFC), being at least 18 years old and being a respected member of the community, and volunteering for the position. The treasurer and secretary also had to be able to read, write, and count. For communities choosing the health sector, the VDC was expanded to include four additional members (2 men and 2 women) chosen by the HFC from among their existing HFC members in order to embed the process in the existing health system structure. In instances where HFCs were defunct or had exceeded their mandate, the health zone authorities were engaged by the project to facilitate new HFC elections. HFCs are mandated by the DRC Ministry of Public Health (MoPH) and their participation in the VDC was considered necessary to ensure the sustainability of projects after the end of *Tuungane*. It is part of the health zone authorities’ responsibility to supervise and support HFCs.

### The Tuungane Community Scorecard (CSC) process

**Fig. 1:**
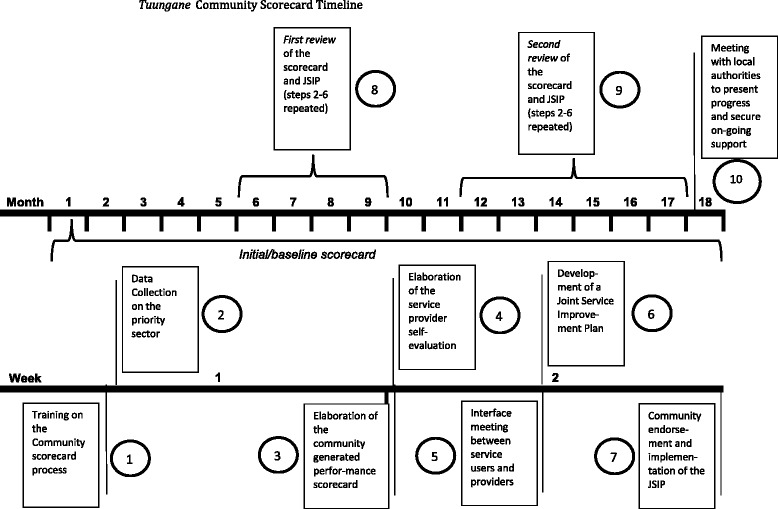
*Tuungane* Community Scorecard Timeline

The CSC involved multiple, participatory steps as shown in Fig. 1. After election of the VDC, VDC members and service providers (head nurse, nurses, and other medical personnel) were trained on the CSC process and data collection (Step 1). The VDC then compared the MoPH standards for health facilities to actual available resources using an input tracking matrix (Step 2). Next, the community generated their performance scorecard, which involved a minimum of 60 community members as well as the VDC and HFC members, and the village leaders. The community participants were a convenience sample of those interested in the process and/or mobilized ahead of time by the Village Chief and the VDC. They were organized into three focus groups (divided into women, men, and the elderly), and at least one third of the community members participating were required to be women. Each focus group generated their own indicators and scores for service delivery performance (for example, cleanliness of the facility, feeling welcomed by providers, or state of infrastructure). In addition, participants provided scores for four standard indicators: access to services, quality of services, engagement of the HFC in financial management, and equal treatment (Step 3). Service providers also developed their own performance indicators and responded to the community-generated indicators and also the four standard indicators (Step 4). Next, there was an interface meeting between the VDC, village leaders, HFC members, services providers, and at least two representatives of each focus group to identify priority issues emerging from the two scorecards, as well as the input tracking matrix (Step 5). The resulting joint service improvement plan (JSIP) included priority actions, such as advocacy towards the health zone for increased personnel or construction of a birthing room (step 6). The JSIP was validated by a general assembly of 60 to 100 community members and later endorsed by line ministry and local government authorities prior to implementation (Step 7). The VDC was responsible for overseeing the implementation of the JSIP using *Tuungane* and community resources (community members’ time and labor and at times materials such as bricks produced by the community). Approximately six months later (corresponding with 50 % completion of the infrastructure project), the first CSC review was conducted (Step 8), followed by a second review at the end of the *Tuungane* project cycle (usually 6–9 months after the first CSC review – Step 9) to gauge progress on the implementation of the JSIP. A few weeks after the second review of the scorecard, VDC representatives from multiple communities presented their respective JSIPs to local line ministries and officials to share progress and secure ongoing support beyond the duration of the *Tuungane* project (Step 10). *Tuungane* staff facilitated the process through the first review in close collaboration with VDC members who took the lead for the second review of the CSC and the meetings with local authorities.

### Most significant change technique

The Most Significant Change (MSC) technique is “a participatory process [that] involves the collection of stories on the most significant change at the field level, and the systematic selection of these stories by panels of designated stakeholders or staff [14].” This method was chosen for program monitoring because of its participatory approach which aligned well with the community-driven focus of the project and to help identify the nature of changes that resulted from the CSC, as perceived by those directly involved in the scorecard process. The MSC technique was selected because of its potential to benefit from the wealth of knowledge and experience acquired by Tuungane staff during the implementation of the project in the field, in a systematic way. For this purpose, the staff were requested to contact beneficiaries who they felt had been highly engaged in the process and would be able to reflect on it, from communities that experienced “significant” changes following the CSC process, instead of collecting feedback from beneficiaries chosen at random. The technique therefore brought to light the most significant changes resulted from the CSC process, identified by staff members and told through the voice of beneficiaries. While not necessarily representative of the CSC implementation as a whole, these contributions allowed for a better understanding of the CSC’s *pathways of change* in the cases significant changes actually materialized.

A total of 125 stories from 79 CSC sites were collected in two rounds for the project; of those, 45 stories were collected from community members and healthcare providers in 25 out of a total of 151 sites that chose the health sector. A group of *Tuungane* field staff (14 of 17 of whom were male) were trained to collect stories using a structured questionnaire that asked what were the most significant changes in service delivery observed by the respondent since the start of the project, and included standard follow up probes, including whether there were any negative effects of the change. Staff were explicitly told to focus on “significant” changes, not “successful” changes. The sampling of the participants by the *Tuungane* field staff was purposeful to ensure inclusion of men and women, healthcare providers, community leaders, VDC and HFC members, as well as other health service users. However, VDC and HFC members were oversampled because of their level of engagement with the project and their ability to speak to how and why changes happened in addition to having witnessed them. Similarly, while the wording of the question did not specifically ask about positive changes, project staff generally selected respondents who they knew had participated actively in the scorecard process and could report changes taking place in their community. Although there was a broader participatory process conducted with project staff to examine the MSC stories and use this to inform program monitoring and implementation, this paper focuses on a separate content analysis conducted on the stories. All stories collected were used in the content analysis.

### Data collection and analysis

There were two rounds of data collection in June-September 2012 and May-August 2013. IRC staff were trained in the MSC methodology and conducted the data collection. Thirteen stories were collected in the first round, and 32 in the second. During the first round respondents were asked to describe the most significant change they had observed since the baseline scorecard, and this sometimes resulted in several changes being mentioned in a single story. In the second round, the interview guide was modified to focus on a single change and probe further into how the change came about. The interview guide was written in French and translated into Kiswahili. Interviews were conducted in Kiswahili and Kihavu. Notes were recorded in French or Kiswahili, and the final write-ups and analysis were conducted in French.

Two of the authors conducted thematic analysis of story transcripts from the education and health sectors for project purposes, using Saturate [15] to code for types of change. Three authors conducted additional analysis focused on the health stories by regrouping codes and stories, and comparing results. The original coding framework was not pre-determined, but was developed using an emergent, iterative approach. These codes were categorized in the subsequent analysis according to domains of health service quality and building blocks of the health system.

Interview respondents were asked if they consented to participate, to the publication of their story, and to have their real name used. All stories were collected as part of routine program monitoring and evaluation. Upon deciding to publish the results, the IRC applied for and received post-hoc ethical review approval from the Catholic University of Bukavu. There was minimal risk to respondents as the material discussed in interviews was not sensitive or personal in nature.

## Results

A total of 45 stories of change were collected in the health sector: 20 from ten CSC sites in South Kivu, and 25 from 15 CSC sites in Katanga. Up to five stories were collected from each CSC site. The profiles of the respondents are presented in Table 1.

**Table 1:** MSC story respondents

	Male (% among males)	Female (% among females)	Total (% of all respondents)
Healthcare provider	6 (23)	1 (5)	7 (16)
Traditional chief	3 (12)	0 (0)	3 (7)
Village development committee member	8 (31)	6 (32)	14 (31)
Health user committee member	6 (23)	8 (42)	14 (31)
Other health service user	3 (12)	4 (21)	7 (16)
TOTAL	26 (100)	19 (100)	45 (100)

### Types of changes

Although not all respondents from the same CSC site cited the same change, most identified similar types of changes. Increases in community participation in the management of the health facility, in particular through greater engagement of health facility committees, and increases in quality of care were recurrent themes. There were several dimensions of quality of care highlighted by these changes, including increased access, improved patient-provider relationships, improved technical performance, and improved maintenance of infrastructure.

#### Increased participation of health facility committees in promoting transparency and improved management

Increased transparency and participation was the most frequently mentioned change. A common theme was the positive shift in the involvement of the HFC in the management of health services or re-activation of dormant HFC. For healthcare providers, changes in the management of their facilities, particularly increased cooperation with the HFC and community, dominated the responses. One healthcare provider described it this way:

*Since the creation of the health center, the [HFC] existed in name only. The members knew nothing about their roles. They were even afraid of approaching the head nurse to share complaints from the community, or even to ask about the status of the medical supplies. Worse, no one had the courage to ask how the center operated. So everything was done without the knowledge of the [HFC], and it was the private domain of the head nurse and his nurses. For the head nurse, the data collected on the sector raised his awareness of the lack of involvement of the [HFC] in the management of the health center. For the president and other members of the [HFC], they realized through the community scorecard process that they were not very active, even during the interface meeting. That is why the president [of the HFC] organized a meeting to be coached and take on more leadership. Since then, [HFC] members play their roles easily, they work closely with the health staff, they are also available to respond to complaints from the community and to raise these at the health center. –Healthcare Provider, Katanga*

#### Improved maintenance of physical infrastructure

One of the responsibilities of the HFC is to mobilize the community to help maintain the health facility and its compound, but in many communities this does not happen. Following the CSC process, some respondents reported greater involvement of the HFC in health facility maintenance, as illustrated below:

*I now notice that there’s a new energy in our community. The [HFC] is present to oversee the cleanliness of our health post and now participates in its management without difficulty. The community is mobilized to work together and is more united that it was two years ago. – VDC member, Katanga*

#### Improved performance

Users had previously been discouraged by the unavailability of drugs and healthcare providers, and instead went to traditional healers or private drug sellers. After the CSC process, some healthcare providers solicited and received support from health zone management teams, other NGOs, and the HFC to address these issues. One health provider described the following changes:

*Now, there is the accounting that we do at the end of each day together with [the HFC]. We plan together and assess our current needs. This new management system has the advantage that we no longer have stockout of drugs and equipment. We have also managed to put in place a rotation system that allows healthcare providers to alternate night and day shifts for service provision, which partially solved the problem of motivation [of healthcare providers who used to feel overworked]… the population has also regained its confidence in modern [medicine] gradually abandoning [shamanistic healers], this was a result of sensitization conducted by the [HFC]. – Healthcare Provider, Katanga*

#### Improved rapport and fairness

Community members reported that healthcare providers were more willing to listen and more respectful in their dealings with users. This increased communication helped create a more welcoming atmosphere at the health facilities. Women often focused on improvements in quality of care, particularly the attitude and behavior of providers, as the most significant change. One HFC member reported these changes after the baseline scorecard:

*…[we see] a warm welcome is reserved for patients by providers. Indeed, the reception given to patients influences healing, we see that now providers demonstrate consideration for, esteem…for their patients… [we see] fair treatment of the sick, that is to say that before Tuungane came, most often in the waiting lines some people were privileged to jump the line for consultations given their influence in society (local authorities, merchants…) and relationships with the nurses (friends, husbands, wives, people close to them…). The most significant change is the warm welcome reserved to patients by nurses, something that has not only strengthened cooperation, the consideration of, and esteem for, but also the healing of the sick. Finally, a good building, equipment, without… a welcoming staff in the facility it leads to nothing because if the impression of the welcome is negative, the rest is irrelevant. – VDC and HFC member, South Kivu*

#### Improved financial access to services

There was increased access to services reported in many communities, primarily because of changes in user fee policies or a reduction in bribes requested from users. This change was mostly one identified by male respondents. In some cases, HFC members and frontline service providers advocated for increased oversight from the health zone management team to encourage the regular payment of salaries for service providers and to dissuade health providers from demanding bribes. Most respondents stated that decreases in user fees or informal payments resulted in increased utilization such that providers did not suffer from decreased income. One HFC member described the situation in his village:

*The situation of our health center before was really catastrophic mainly because there was a very high user fee for receiving health care services. This was due to the fact that the management of the health center was done exclusively by the head nurse. He, with his staff, did what they wanted. This is what has been done to resolve this situation: we, members of the [HFC], with the VDC members, have organized a meeting with the frontline service providers to discuss a reduction in the health care cost. The head nurse told us that it is very difficult to reduce user fees, and yet most nurses are not registered by the State. Our resolution to this meeting was to send a correspondence to the health zone. The letter was signed by the president of the VDC and [HFC], as well as the head nurse and the local authority [Village Chief]. In the letter, all the difficulties of the health center which could be addressed by the health zone were presented, among others the construction of a nutrition [hangar], the lack of registration of nurses by the State, and the lack of medical supplies. After the change, user fees were significantly reduced to approximately 0.5 USD for a child, and approximately 1 USD for an adult. –HFC member, South Kivu*

### Mechanisms of change

**Fig. 2:**
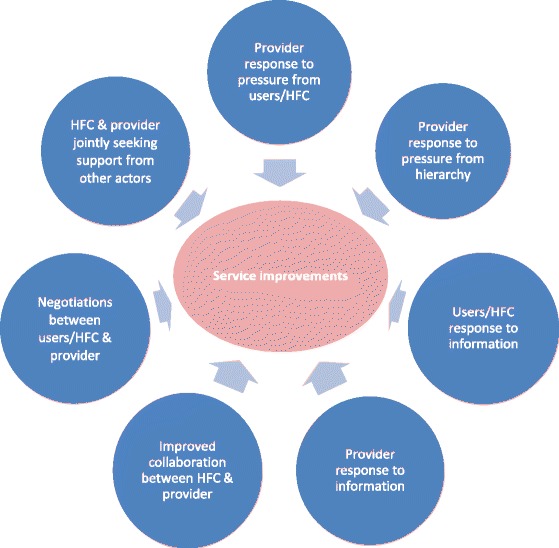
Conceptual framework of mechanisms influencing changes in the health system

Most stories report a change in the relationship between the HFC and healthcare providers. They offer examples of committee members acting collectively to improve service delivery, applying pressure on their healthcare providers, and advocating to health zone level management and external actors. Figure 2,^1^ represents several, non-mutually exclusive mechanisms of change observed in the stories. There may have been multiple changes and pathways in each village, and there may be other mechanisms that have not yet been documented. Examples of more prominent mechanisms are described below.

#### Healthcare provider response to information and pressure

Some stories indicate that healthcare providers changed their behavior in response to social or hierarchical pressure, economic interests, concern for the community’s well-being, or a combination of these. For example, one VDC and HFC member reported during the course of the CSC that the head nurse became aware that some health facility staff were not respecting posted user fees, and were asking for bribes or additional payments. The head nurse felt that this would discourage users from coming to the facility. He organized a meeting with his staff and community members to demand that his staff respect payment procedures while asking the HFC members to encourage users to frequent the facility. Because the community was informed of this initiative, they reported any staff who asked for bribes. In another village the VDC, the HFC and the village chief were able to convince the nurse at their health post to return drugs that he had stolen.

#### Improved collaboration between HFC and healthcare providers

Many HFC and healthcare providers became better informed of their roles and responsibilities. Empowered HFC were able to work with healthcare providers who were willing to make changes. In one village a health user reported that the HFC started meeting monthly with the healthcare providers to troubleshoot problems, including user complaints and management of drug stocks, and information from these meetings was shared with the population. The improved relationship between HFC and healthcare providers led to a decrease in user fees in another village, which helped increase utilization of services.

#### Users and healthcare providers jointly seeking support from other actors

As described above, some committees and providers sought support from the health zone level for fee reduction. Other examples of collaborative efforts include committees who sought assistance from non-governmental organizations to improve services or asked the health zone management team for training for healthcare providers.

## Discussion and limitations

Fragile and conflict affected states do not provide a context conducive to civic engagement [11]. The findings demonstrate pathways through which the CSC process can improve accountability and influence the quality of health services in one such setting. This includes increased participation of health facility committees in promoting transparency and good management, improvements in physical infrastructure, improved performance of providers, better rapport between providers and patients, and increased financial access to services. Several of these changes are interrelated, for instance, improved infrastructure may make providers happier about working, which inclines them to be kinder to patients, and more willing to negotiate user fees. The increased participation of HFC may also contribute to improved conditions for providers and improve their attitudes toward community members. All of these factors may improve access to and equity of services. Although there is limited literature on the effectiveness of CSC specifically, the finding of improved access is consistent with literature on community participation in health and HFCs [8]. While the data presented here do not objectively measure outcomes such as increases in utilization or decreases in drug stock-outs, they show how community participation in health can produce improvements through facilitating flows of information, increasing collaboration, and supporting user demands regarding their entitlements.

### Effects of improved access to information

In CSC interventions in India and Madagascar, a change in the providers’ responsiveness to users was a result of improved channels of communication and mutual understanding [12]. In Uganda, Nyqvist, et al. (2014) also found that a participatory intervention that included both participation and information on staff behavior had short and long-term effects, while interventions that did not include information on staff behavior had no impact on quality [16]. The *Tuungane* CSC process provides information on MoPH standards and entitlements, as well as wider community perceptions of service delivery performance in a way that is unprecedented in most of the targeted communities. By introducing a discussion on broader service delivery issues than they might otherwise have considered, the CSC allowed service users and providers alike to gain access to information that they otherwise would not have had and to consider and act on critical service delivery issues such as staffing, user fees and patient-provider relationships, in addition to the common focus of infrastructure. Sharing information on services publicly may have compelled individuals with responsibility in the community to act.

### Joint problem solving

In a fragile state where line ministries may lack the capacity or will to provide adequate oversight to peripheral parts of the health system, the impact of the CSC on governance is particularly important. Both service providers and HFCs embraced their mandated roles and responsibilities in a process where the VDC members represented the broader community, and where all participants were informed of MoPH standards. Committee members were able to monitor adherence, and seek redress from higher levels. In the DRC, health zone management teams receive no direct financial support from the MoPH, other than their salaries, and these are low and irregular, if paid at all. Lacking resources to pay for fuel if they even have a vehicle or motorcycle to conduct supervisions, they may rarely visit peripheral health facilities. Through the CSC, communities have demonstrated how they can empower HFCs to monitor and take action to improve health facility performance whether or not they receive reinforcement from the health zone management team, at least for those problems that can be solved at the very local level, similar to findings from Bjorkman and Svensson in Uganda [17]. It is important to note how the context in which this project took place may have affected the results [18]. Although the DRC has not devolved management of health services officially, the weak presence of the state in remote areas may have made it easier for these local changes to happen.

### Perspectives and participation of different stakeholders

Given the lack of community engagement in the management of health facilities prior to the project, it is not surprising that for providers the increased participation of community members in health facility management was the most notable change. That more women highlighted the changes in quality of care including the attitudes and behaviors of providers, is also not surprising given that women frequent health facilities and use services more often than men, especially to accompany their children. The economic considerations of access may have been more noticeable a change for men because in this context they tend to dominate household decision-making around economic resources. Nevertheless, women and men’s responses might have been two reactions to the same phenomenon of nurses abusing their power. However, women would rather highlight that the nurses’ “unwelcoming behavior” resulted in them (or their children) not having access to services; men on the other hand might have shown more sensitivity to issues related to power dynamics within the village, and illicit gains. We did not observe any variation in response by membership in HFC or VDC. The Health Service Delivery Community of Practice [19] recently put forward 12 recommendations for renewing the health district in Africa in order to advance universal health coverage [20]. These included a greater role for individuals, households, and communities as “co-producers of their own health” through empowerment, freedom, and citizen voice; and a more flexible, open approach to the district health system to allow for localized responses to the population’s needs. Through the CSC process, communities adapted policies to their context and negotiated local solutions to improve their health services. More than one community was able to increase access to services by negotiating changes in the health facility user fee policy. Although there is clear evidence of the potential positive effects of fee exemptions on health outcomes, top-down policies implemented without careful planning and engagement of stakeholders have encountered many challenges such as confusion or lack of information among the population regarding overlapping policies and inadequate measures to overcome equity issues [21]. Where user fees were modified in *Tuungane*, the process was negotiated between the parties most directly affected by the change - community members and providers - which is more likely to lead to a mutually acceptable result.

The literature suggests that the selection and composition of HFCs can have an influence on their impact, with lack of transparency being a potential challenge [22]. Although the HFCs were not systematically re-elected under *Tuungane* to ensure that they represented the community, the participation of the freely elected VDC and wider community in the process ensured a certain level of transparency and oversight in the implementation of the JSIP.

### Role of the community grant

While the CSC directly affected power imbalances between users and healthcare providers through information sharing, as part of the *Tuungane* program the community also received a grant of 24,000 USD for implementation of the service improvement plan. This plan was managed by the VDC which included members of the HFC, giving them control and oversight over an enormous financial resource that was also valued by providers. In order to access this resource, healthcare providers needed to work with the VDC, and this may have been a motivation for them to improve relationships with VDCs as working conditions are important to providers and the grants were generally used to improve this However, given the broad range of changes elicited through the project, particularly in terms of the relationships between users, health workers and HFCs, it is reasonable to suggest that some of these changes would continue to manifest themselves beyond the life of the project and the community grant. Particularly in terms of information gained by users about their entitlements- although the sums would not be as large as the grant, because of the cost recovery system and lack of salary paid by the state, users still have leverage over providers’ income if they perceive a drop in quality of services.

### Assumptions about state-society relations at local level

The types of collaboration illustrated through the MSC stories has brought to light an observation that was not explicit in the original theory of change, that often the disconnect at the local level was not between community members and providers, but between them collectively and higher level authorities. That is, the conceptual divide between citizen and state was not necessarily so useful when examining community members and local healthcare providers. Local healthcare providers live in these communities and may have social incentives to get along with community members, and in most cases they were not even on the state payroll, as is the case across much of the DRC. In some cases when providers and communities came together, they were more confident and more capable of demanding entitlements or support from zonal health authorities. As is highlighted in the conceptual framework of mechanisms influencing changes in the health system, healthcare providers are not always motivated by self-interest and change does not always come about through confrontation with users. While the power differential between health workers and users cannot be underestimated, it is also true that the status quo is often maintained, not necessarily by a desire on the part of health workers to hold on to privileges and exploit users, but often just by poor information flows and a lack of understanding of shared needs and priorities. In addition, the CSC process demonstrated that top-down health system accountability was not the only mechanism for improvement of services, and that changes could happen at the local level through information sharing, without higher level state intervention or punitive measures. In line with the original theory of change though, responsiveness of providers was key to many of the mechanisms and types of changes observed.

### Limitations

There are some limitations to the data presented. HFC members were the source for 31 % of the stories (14 out of 45), which may have biased the types of changes reported or the roles they played in these changes. Only 16 % (7 of 45) stories were from users who were not a VDC or HFC member or community leader. At the same time, the objective of the collection of MSC was to explore what kinds of changes can result from the CSC process and what the mechanisms of change were. As the average service user did not participate in all steps of the CSC process, they would not have as much insight into the mechanisms of change, particularly around the changes in governance of health facilities. VDC and HFC members were better placed to understand the process through which the intervention stimulated change and comment on how changes occurred. In addition, the stories of change were conceived as part of routine project monitoring and were not originally intended for research.

The scope of this paper focuses on the content of the stories and not the entire MSC process and its influence on program implementation. There are strengths and weaknesses to using this methodology for collection of qualitative data. Decisions about participants were made by field staff with the intention of identifying “significant” changes, and respondents were also asked about any negative effects of the changes. This is very useful for explaining how changes unfolded when the program worked as intended, but does not explain failures of the CSC to stimulate desired outcomes. As such we can expect that many participants would solely focus on successful stories. As the purpose of the process was to explore mechanisms of change, the selection of respondents necessarily focused on communities that had experienced change, rather than communities that did not observe changes. Also, stories were collected by IRC staff, which may also have influenced the responses if respondents felt that this could affect continued support from the IRC.

Finally, the perspectives of *Tuungane* staff on accountability have changed over the lifetime of the project. Observations of how change is taking place suggest that a collaborative approach to accountability is just as likely as a confrontational approach in the local DRC context. Our initial assumption, and the one that is often put forward when discussing accountability, is of users needing to reign in the formal power of corrupt or inept health workers. However, program staff saw more examples of what Booth (2012) describes as collective action problems^2^ on the supply and demand sides that need to be overcome [23]. Accountability therefore becomes about efforts among users and health workers and between them to collectively solve problems that plague local health services.

## Conclusions

This study focused on examining changes perceived as significant by the staff and the beneficiaries involved in data collection, and showed some of the mechanisms by which CSC can improve the functioning of local health systems in fragile and conflict-affected settings by providing information to users and providers and encouraging them to engage in making health services more responsive to their needs. It brings into question whether frontline healthcare providers are part of the state or society, depending on the context and perspective of the observer, and how this influences how they act and respond to users. In the setting of the DRC where the central government has limited influence on many aspects of what happens in the periphery, the divisions between frontline healthcare providers and community members can be bridged by facilitating space for interface, exchange, and collaboration. Further studies should include both qualitative and quantitative data to understand the objective effects of CSC, the mechanisms by which they work in each context, and whether changes are sustained over time. Also, given the limited evidence on social accountability tools such as CSC, program implementers should carefully design their monitoring and evaluation systems to ensure data is collected for the purpose of future evaluation. This would help researchers to rigorously assess the impact of such tools and better understand how and why they work.

## Abbreviations

CDR: Community-driven reconstruction
CSC: Community scorecard
DfID: UK Department for International Development
DRC: Democratic Republic of Congo
HFC: Health Facility Committee
IRC: International Rescue Committee
JSIP: Joint Service Improvement Plan
MoPH: Ministry of Public Health
MSC: Most Significant Change
VDC: Village Development Committee

## Footnotes

1 This figure is adapted from a briefing paper by Leni Wild and Marta Foresti (2012): ‘More than just ‘demand’: Malawi’s public-service community scorecard. See: http://www.plan-uk.org/resources/documents/community-scorecard-malawi-odi-project-briefing/.

2 Collective action problems can occur in situations whereby multiple individuals would all benefit from a certain action but such action has an associated cost making it implausible that any one individual can or will undertake and solve it alone. These problems are overcome when a coordination mechanism is put in place that allows these costs to be shared.
